# The effect of retrogradation time and ambient relative humidity on the quality of extruded oat noodles

**DOI:** 10.1002/fsn3.1596

**Published:** 2020-04-28

**Authors:** Rui Dong, Qianwen Niu, Kailong Zhang, Xinzhong Hu, Yu Bu

**Affiliations:** ^1^ College of Food Engineering and Nutritional Science Shaanxi Normal University Xi'an China

**Keywords:** oat, retrogradation, starch

## Abstract

Commercial process of oat noodles was mainly hindered by its lack of gluten and difficulty in forming dough. Extrusion could be an effective method to produce oat noodles by forming network of gelatinized starch, and noodle quality could be improved by regulating the retrogradation process. In this study, we produced oat noodles by two‐step extruding and investigated the effect of retrogradation treatment (retrogradation time and ambient relative humidity) on noodle properties. At each corresponding ambient relative humidity (RH), the starch crystallinity and enthalpy value increased, while setback value decreased, as well as noodle cooking loss was significantly improved as retrogradation time increased to 48 hr, and then decreased at 72 hr. At the same retrogradation time, the starch crystallinity, setback, and enthalpy value decreased to RH70% and then had a slight rise at RH80%, while noodle cooking loss with reversal trend. The retrogradation time of 48 hr and ambient RH of 60% could be an optimum treatment for effectively improving extruded oat noodle quality. Furthermore, multivariate data analysis indicated that samples at the same ambient RH tended to be clustered together. This study could provide basic knowledge for controlling processing condition of the extruded oat noodle.

## Graphical abstract

Quality of extruded oat noodles was promoted by retrogradation treatment. Optimal retrogradation condition, including retrogradation time and ambient relative humidity, was investigated through studying the relationship between noodle quality and starch retrogradation.

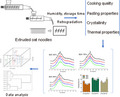

**Highlights**

1. Ambient RH and retrogradation time affect oat noodle quality and starch properties.
2. Retrogradation conditions improved noodle quality by regulating starch crystalline.
3. PCA and cluster analysis were used to optimize and verify noodle processing.

## 1 INTRODUCTION

Oat is recognized as a healthy food due to its physiological functions, such as its hypoglycemic effect, balancing gut microbiota, and lowing cholesterol (Daou & Zhang, 2012; El Khoury, Cuda, Luhovyy, & Anderson, 2011; Martinez‐Villaluenga & Penas, 2017). Conventional oat products, such as oat flakes, are very popular among consumers (Rasane, Jha, Sabikhi, Kumar, & Unnikrishnan, 2015). However, the demand for new and innovative oat products grows rapidly. Noodles are a kind of typical staple food and win great popularity in Asia. Therefore, oat noodles can be an ideal oat product with a great market. The development and investigation of oat noodles have attracted numerous attention in both industry and academia (Majzoobi, Layegh, & Farahnaky, 2014). Unlike wheat, oat is lack of gluten and accordingly is hard to form similar network in oat dough. This limitation is a major obstacle to its commercial process (Witczak, Ziobro, Juszczak, & Korus, 2016). In recent years, extrusion technology has been successfully applied to produce nongluten noodles (Dissanayake & Jayawardena, 2016; Shiau, 2010). The raw material is subjected to strong kneading, and its large proportional starch is gelatinized during extrusion, thereby promoting the formation of network (Shiau, 2010). Oat noodles can be prepared by this technique, and it requires reboiling or soaking in hot water before eating (Reungmaneepaitoon, Sikkhamondhol, & Tiangpook, 2006). However, extruded oat noodles are faced with high cooking loss and poor eating quality after boiling. Furtherly, regular one‐step extrusion could not produce oat noodles with satisfying cooking quality, so a second extrusion was introduced to press the intermediate product, which could give the noodle uniform and compact shape, which could meet with consumer's requirements.

With regard to the nongluten food, starch plays an important role in its processing and eating qualities (Witczak et al., 2016). Regulating or changing starch structure, that is, amylose and amylopectin, crystalline structure, and granular structure, can drastically affect noodle qualities (Li, Dhital, & Wei, 2017). Starch retrogradation is a process that gelatinized starch molecules rearrange and form a more ordered structure, which contribute to the increase of crystallinity and endow the products’ apparent quality with firmness and rigidness (Li et al., 2017; Wang, Li, Copeland, Niu, & Wang, 2015). This process is influenced by several factors, such as storage temperature, storage time, moisture content, and additives (Aguirre, Osella, Carrara, Sánchez, & Buera, 2011; Bello‐Pérez, Ottenhof, Agama‐Acevedo, & Farhat, 2005; Patel & Seetharaman, 2010). Several studies have found drying temperature had no significant effects on the cooking loss of rice noodles, and some other researches have got similar results (Aktan & Khan, 1992; Lee, Woo, Lim, Kim, & Lim, 2005). In general, starch retrogradation possesses two processes, short‐term and long‐term retrogradation. Short‐term retrogradation can be very quickly, usually completed within several hours after gelatinization; this process is dominated by amylose rearranging (Xiong, Li, Shi, & Ye, 2017). In contrast, long‐term retrogradation usually takes several days and is mainly the rearrangement of amylopectin (Krystyjan, Adamczyk, Sikora, & Tomasik, 2013). Water also plays a crucial role in the retrogradation process. Within a certain range of water content, the increase of water can promote starch crystallization, because amylopectin can move more quickly under ample water content, while excess water leads to an inhibition of crystallization owing to its dilute effect making amylopectin with more mobility and rearranging more difficult (Ding, Zhang, Tan, Fu, & Huang, 2019).

Different retrogradation treatment condition resulted in different retrogradation phenomenon and affected the noodle texture significantly (Mariotti, Iametti, & Cappa, 2011). Regulating the environmental storage condition is an effective way to promote starch retrogradation for its easy handling, energy saving, and avoiding of external additives. In this study, we aimed to investigate the extruded oat noodle starch retrogradation and the noodle qualities under different ambient relative humidity and storage time, exploring the effect of regulating storage condition on starch structure and noodle qualities. This study can provide a theoretical basis for the development of commercial oat noodles.

## 2 MATERIALS AND METHODS

### 2.1 Preparation of extruded naked oat flour noodle

**Figure 1:**
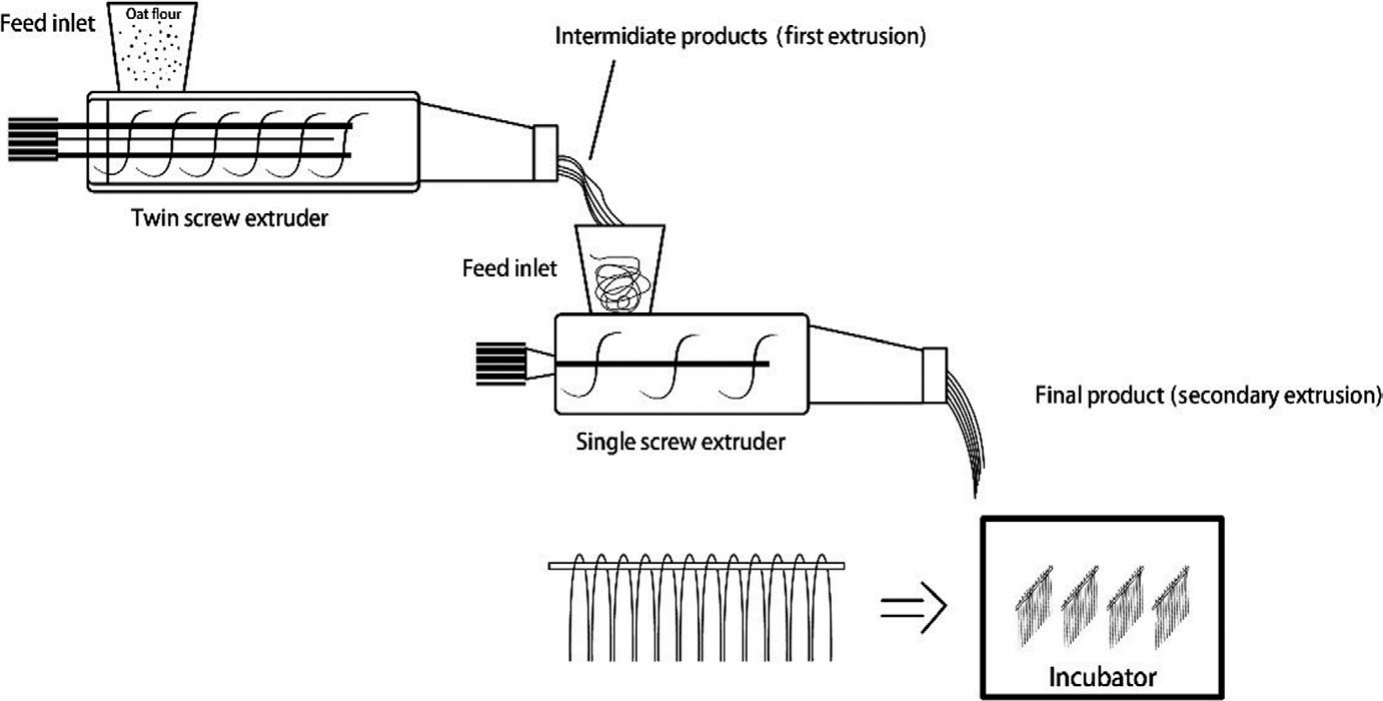
Illustration of extruded oat noodle processing

The oat flour was obtained from Mengye Company in Inner Mongolia, which was milled from Chinese naked oat (moisture content 13%). The extruded oat flour noodles were produced using a factory‐scale twin‐screw extruder (YMP, Dayi, Jinan). The temperatures of barrel zones from I to β were 70°C, 80°C–100°C, and 140°C, respectively. Water content of the oat flour was adjusted to 38% prior to extrusion. The feed rate and screw speed of the first step extrusion was consistently kept at around 60 kg/hr and 300 rpm while the second step extrusion was a one screw extruder which ran at a speed around 200 rpm without heating process. Sample was extruded twice to produce final oat noodle product. The first extrusion was to pregelatinize the starch, and the second extrusion was to shape the noodles. Producing process can be illustrated in Figure 1.

After extrusion, the noodles were collected and retrograded by regulating the retrogradation time and ambient relative humidity (RH) in an incubator (HWS, Taisite). The retrogradation treatment was carried out for 12, 24, 48, and 72 hr at 25°C with the ambient RH of 60%, 70%, and 80%, respectively. All the samples were collected and transferred to a cryogenic refrigerator (DW‐40L278, Haier, Qingdao) at −40°C once each retrogradation treatment was completed. The sample moisture content was determined by the method GB 5009.3‐2016 (Chinese national standard for food safety: determination of moisture content in food). The samples were kept in the refrigerator until analysis. Samples were freeze‐dried for pasting properties test, X‐ray diffraction (XRD) measurement, and thermal properties test.

### 2.2 Noodle cooking quality test

Noodle cooking loss was determined following the AACCI approved method 66–50 (AACC, 2000). Briefly, 20 g of noodles was accurately weighed and then boiled in 1,000 ml distilled water according to its optimum cooking time. After cooled to room temperature, the supernatant was collected and diluted to 1,000 ml with distilled water. 100 ml of the noodle soup was transferred into a beaker and then heated to evaporate. After most of the water was evaporated, another 100 ml of the supernatant was transferred to the beaker and was evaporated to near dry. Then, the beaker was placed in an air oven and heated to a constant weight at 105°C. The residue was weighted and reported as a percentage of the initial sample. The cooking loss was calculated according to the following equation.

$$\text{Cooking}\;\text{loss}\;=\;5M/\left[G\;\times \left(\text{1}\;-\;W\right)\right]\;\times \;\text{100}\%,$$

where M is the dry matter weight of the supernatant, W is the moisture content of the noodles, and G is the fresh sample weight.

### 2.3 Pasting properties

Pasting properties were determined using a Rapid Visco Analyzer (RVA‐TM, Sweden) according to AACC approved method 76‐21 (AACC, 2000). 4.0 g of flour (with a moisture of 14%) was slurried in 25 ml of distilled water. Parameters including peak viscosity (PV), trough viscosity (TV), and final viscosity (FV), and setback (SB, FV ‐ TV) were recorded.

### 2.4 X‐ray diffraction (XRD) measurement

Starch crystalline structure was analyzed by an X‐ray diffractometer coupled with a Cu‐Kα radiation detector (D/Max2550VB+/PC, Rigaku Corporation) at 40 kV and 40 mA. Freeze‐dried noodle sample was milled and detected with a step size of 0.02 and a scanning rate of 4°min^−1^ from 5° to 45° (Zeng et al., 2015). The relative crystallinity was obtained by using Jade software 5.0 (Materials Data Inc.) to calculate the percentage of the peak area to the overall diffractogram area.

### 2.5 Thermal properties

Thermal properties of samples were carried out on a differential scanning calorimeter (Q600 SDT, TA Instruments). Approximately 2 mg of sample was weighed and placed into aluminum pans, and 2 μl of distilled water was added. The suspension was equilibrated at room temperature overnight prior to test. Samples were heated from 20 to 95°C at a rate of 10°C min^−1^ (Qiu et al., 2017). Onset (T_o_), peak (T_p_), and final temperatures (T_c_), as well as gelatinization enthalpies (Δ*H*), were obtained from the thermograms by the DSC data recording software.

### 2.6 Statistical analysis

The data were presented as mean ± SD. All measurements were carried out with three replicates. Significance was carried out using SPSS software (version 16.0, SPSS Inc.) by one‐way analysis of variance (ANOVA) with Duncan's method.

## 3 RESULTS AND DISCUSSION

### 3.1 Cooking quality

**Figure 2:**
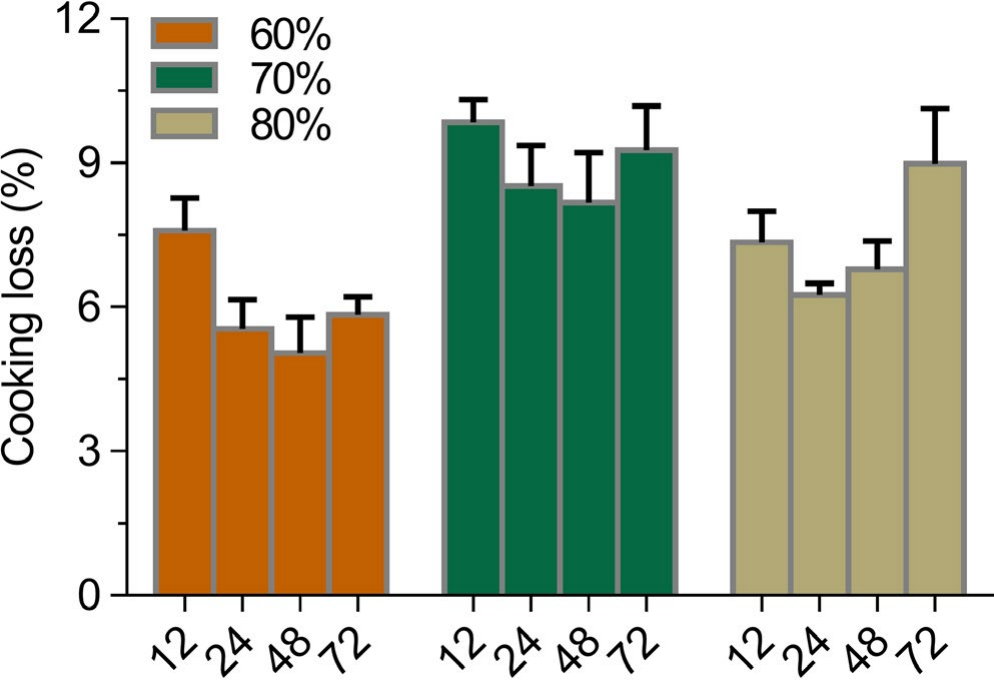
Sample cooking loss under different retrogradation treatment

Cooking loss reflects the cooking resistance of noodles, and it is also an essential criterion for evaluating noodle cooking quality (Chillo, Ranawana, & Henry, 2011). Noodles without retrogradation treatment have a much higher cooking loss than those been treated. As demonstrated in Figure 2, varied retrogradation treatments caused predominant differences in cooking loss. When the RH was constant and retrogradation time was within 48 hr, the cooking loss decreased with time extension and was lowest at 48 hr, whereas a further increase occurred at a longer storage time of 72 hr. Under each ambient RH, the minimum cooking loss was observed at 48 hr, which was 5.04% (RH 60%), 8.17% (RH 70%), and 6.25% (RH 80%), respectively. Cooking loss varied at different ambient RH under varied retrogradation time. The minimum cooking loss was observed at RH 60% under the same retrogradation time, which was 7.34% (RH 80%‐12 hr), 5.54% (RH 60%‐24 hr), 5.04% (RH 60%‐48 hr), and 5.84% (RH 60%‐72 hr), respectively. Cooking loss presented an increased trend as ambient RH rose.

Extrusion processing caused oat starch gelatinizing and formed a network to develop dough, which assisted the structural maintenance of noodles when cooking (Witczak et al., 2016). Time and ambient RH affected the starch crystallinity. The improved cooking quality could be ascribed to the increased crystallinity of starch after retrogradation, which enabled a more compact and rigid network and therefore prevented components leaking outside the network (Li et al., 2017). However, the increase of cooking loss at further extending time was associated with the reversibility of starch crystallization, which conversely resulting in an incompact and week network (Ambigaipalan, Hoover, Donner, & Liu, 2013). In this study, the optimum cooking quality was observed at 48 hr with an ambient RH of 60%, which could be owing to the highest starch crystallinity.

### 3.2 Pasting properties

RVA parameters are summarized in Table 1. SB value reflects retrogradation tendency of amylose. In general, higher SB values show a greater tendency for retrogradation (Ambigaipalan et al., 2013; Zhang et al., 2018). The highest SB values observed at each RH were 447 cP (60% RH‐48 hr), 274 cP (70% RH‐48 hr), and 388 cP (80% RH‐24 hr). As time further prolonged, SB value declined, indicating that the retrogradation level of noodle starch reached a peak and then gradually fell down. This could also be well understood that the whole retrogradation treatment period is a combination of short‐term and long‐term retrogradation. During short‐term retrogradation, amylose recrystallization dominated the retrogradation process, while amylopectin came to dominate the long‐term retrogradation and some short chains were a reversible process (Chen, Ren, Zhang, Tong, & Rashed, 2015).

**Table 1:** Pasting properties of naked extruded oat noodles

Retrogradation treatment	PV/cP	TV/cP	FV/cP	SB/cP
RH 60‐12 hr	1352.00 ± 9.90e	364.00 ± 5.66c	708.50 ± 3.54cd	344.50 ± 2.12c
RH 60‐24 hr	1660.00 ± 7.07cde	489.50 ± 0.71b	925.00 ± 0.01ab	435.50 ± 0.71a
RH 60‐48 hr	2430.00 ± 50.91a	562.50 ± 13.44a	1009.50 ± 17.68a	447.00 ± 4.24a
RH 60‐72 hr	2195.00 ± 497.80ab	475.00 ± 73.54b	856.00 ± 113.14b	381.00 ± 39.60b
RH 70‐12 hr	1469.00 ± 24.04de	241.00 ± 2.83fg	468.00 ± 2.83gh	227.00 ± 0.01fg
RH 70‐24 hr	1558.00 ± 42.43cde	275.50 ± 0.71ef	523.00 ± 0.01fg	247.50 ± 0.71ef
RH 70‐48 hr	1610.50 ± 45.96cde	329.00 ± 5.66cde	603.00 ± 9.90ef	274.00 ± 4.24de
RH 70‐72 hr	1241.00 ± 386.08e	201.00 ± 41.01g	394.50 ± 74.25h	193.50 ± 33.23g
RH 80‐12 hr	2114.50 ± 62.93ab	335.00 ± 18.38cde	631.00 ± 28.28de	296.00 ± 9.90d
RH 80‐24 hr	1978.00 ± 1.41bc	353.50 ± 2.12cd	741.50 ± 0.71c	388.00 ± 2.83b
RH 80‐48 hr	1996.00 ± 130.11abc	320.00 ± 5.66cde	628.50 ± 9.19de	308.50 ± 3.54cd
RH 80‐72 hr	1823.50 ± 72.83bcd	294.50 ± 13.44def	568.00 ± 21.21ef	273.50 ± 7.78de

Note

PV, TV, FV, and SB are peak viscosity, trough viscosity, final viscosity, and setback.

Data with different letters in the same column mean significant difference (*p* < .05).

At the same retrogradation time, SB value of the samples shows a decreased tendency as the ambient RH increased from 60% to 80%. The highest SB values were observed at an ambient RH of 60%, which were 344.50 cP, 435.50 cP, 447.0 cP, and 381.00 cp, respectively. Higher ambient RH promoted the moisture diffusion in noodle sample, which affected the starch retrogradation accordingly (Johnson & Mauer, 2019). The increase of moisture accelerated movement of amylopectin and increases the probability of forming crystals, therefore resulting in an increased retrogradation degree. However, excess moisture reduced the possibility of starch molecule interacting due to the dilute effect, which in turn reduced the degree of starch retrogradation (Ding et al., 2019).

### 3.3 Thermal properties of samples

The transition temperatures (To, Tp, Tc) and melting enthalpies of samples under different RH and retrogradation time are summarized in Table 2. In the same ambient RH, the enthalpy was significantly increased as storage time prolonged within 48 hr, whereas further storage time causes a slightly decrease of enthalpy. The enhancement of enthalpy indicated more energy was required to melt the reassociated amylopectin crystallites (Cooke & Gidley, 1992; Shi, Chen, Yu, & Gao, 2013). As time increased, the gelatinized starch molecules tended to rearrange and form more ordered and stable structures. However, a slight drop of enthalpy at 72 hr could be ascribed to reversible crystallization process of amylopectin. The short‐term retrogradation of starch usually occurs with a few hours, which is mainly dominated by amylose recrystallization and is unreversible, whereas the long‐term retrogradation is dominated by amylopectin recrystallization and is reversible (Miles, Morris, & Orford, 1985), which was because that the amylopectin recrystallization of shorter branch of (DP14 ‐ 18) has less stability than amylose crystal (Karim, Norziah, & Seow, 2000).

**Table 2:** Differential scanning calorimetry results of extruded oat noodles

Retrogradation treatment	To/°C	Tp/°C	Tc/°C	Δ*H*/J/g
RH 60‐12 hr	48.11 ± 2.16ab	56.17 ± 0.28b	62.61 ± 1.39a	0.52 ± 0.03ef
RH 60‐24 hr	44.82 ± 1.19cd	55.20 ± 0.71bc	62.08 ± 1.26a	0.59 ± 0.02de
RH 60‐48 hr	46.36 ± 1.89abcd	54.38 ± 1.63bc	61.73 ± 2.31a	1.10 ± 0.11a
RH 60‐72 hr	46.65 ± 0.78abcd	54.83 ± 1.16bc	61.90 ± 1.39a	0.95 ± 0.01b
RH 70‐12 hr	44.74 ± 1.48cd	55.02 ± 0.38bc	62.96 ± 1.12a	0.39 ± 0.06g
RH 70‐24 hr	44.92 ± 0.50bcd	54.28 ± 0.63bc	60.70 ± 0.41ab	0.46 ± 0.01fg
RH 70‐48 hr	44.14 ± 0.38cd	54.85 ± 0.18bc	63.14 ± 1.26a	0.52 ± 0.04ef
RH 70‐72 hr	43.97 ± 0.98d	53.65 ± 0.64c	60.45 ± 1.06ab	0.48 ± 0.02f
RH 80‐12 hr	47.41 ± 1.53abc	54.42 ± 0.03bc	58.19 ± 1.36b	0.39 ± 0.21g
RH 80‐24 hr	43.75 ± 1.48d	53.78 ± 0.95c	60.79 ± 0.94ab	0.76 ± 0.01c
RH 80‐48 hr	44.39 ± 1.65cd	54.32 ± 1.48bc	62.34 ± 1.90a	0.63 ± 0.01d
RH 80‐72 hr	48.54 ± 0.75a	60.76 ± 1.06a	60.76 ± 1.06ab	0.59 ± 0.01de

Note

To, Tp, Tc, and Δ*H* are onset temperatures, peak temperatures, final temperatures, and gelatinization enthalpies.

Data with different letters in the same column mean significant difference (*p* < .05).

Water content could also produce a great influence on starch melting enthalpies. As shown in Table 2, the enthalpies exhibited a decreasing tendency as ambient RH increased from 60% to 80% at each corresponding time. Higher environmental RH enabled oat starch to absorb more water, which could make amylopectin move more quickly and accordingly contribute to its retrogradation process ((Johnson & Mauer, 2019). However, excess water had a dilute effect on the starch molecule and appeared as starch with more mobility, which made the crystallization more difficult, therefore inhibiting the retrogradation process, and produced a less ordered and stable starch molecule (Ding et al., 2019). The enthalpies were consequently decreased. This result indicated that the ambient RH of 60% and storage time of 48 hr could produce the most stable crystallites.

### 3.4 Crystal structure

**Figure 3:**
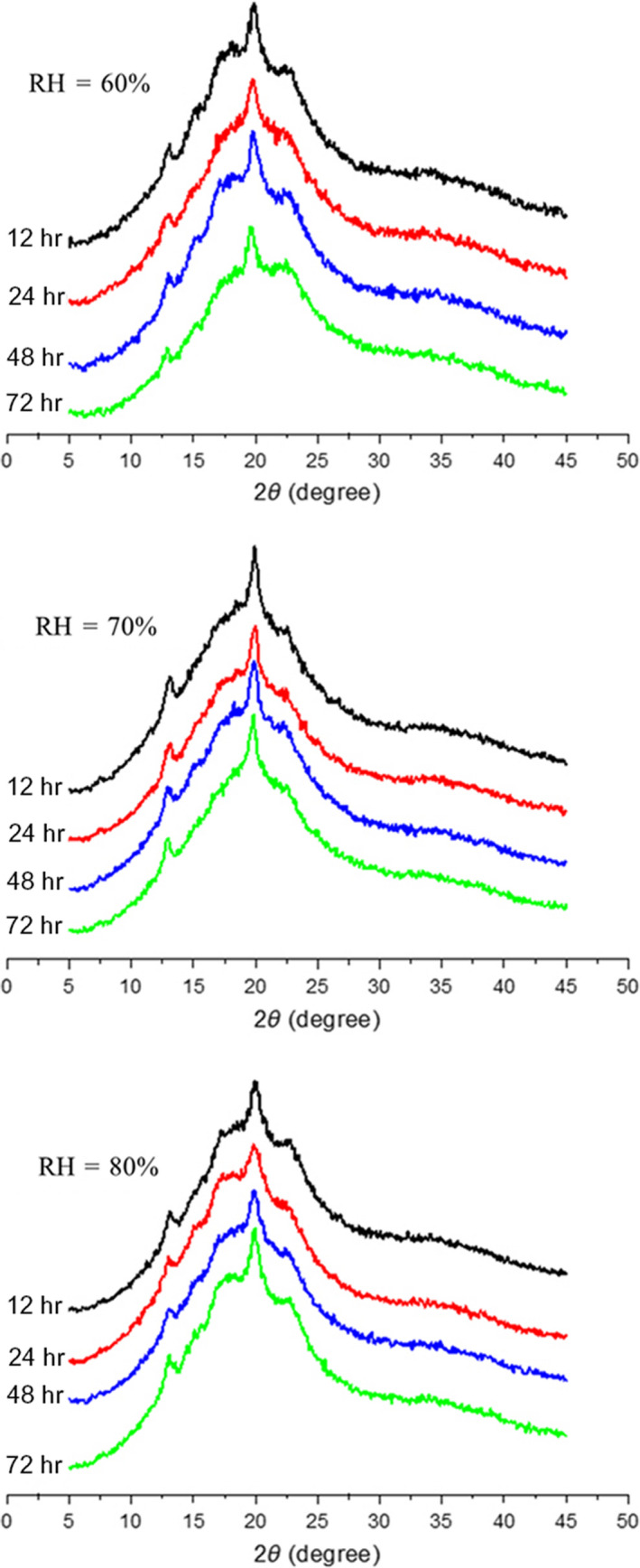
X‐ray diffraction patterns of oat noodles under different RH and retrogradation time

X‐ray diffraction can well reflect the starch crystallinity (Figure 3). As demonstrated in Figure 1, the noodles with all treatments showed diffraction peaks at 2θ of 13.8°, 17.5°, and 19.5°, respectively, indicating a typical B‐type structure (Zobel, Young, & Rocca, 1988) and a V‐type structure (Hoover, Smith, Zhou, & Ratnayake, 2003). This could be ascribed to the cooling process and retrogradation of gelatinization starch during storage. The retrogradation starch showed an obvious difference in the relative crystallinity as the storage time increased. After extrusion under high pressure and high temperature, V‐type crystalline was formed because of the starch and lipid complex. The most intensive peak around 20° was observed in each sample, which indicated that V‐type structure was intensified by complex of starch and lipid (Hoover et al., 2003). For the noodles at the same ambient relative humidity, the relative crystallinity gradually increased and reached peak at 48 hr (Table 3). For short‐range molecular order, the amylose retrogrades very quickly, while long‐term retrogradation could take several days to complete amylopectin rearranging (Biliaderis, 2009). In this study, the retrogradation treatment process on samples in all ambient RH had completed within 48 hr.

**Table 3:** Relative crystallinity of extruded oat noodles after retrogradation treatment

Sample	RH 60‐12 hr	RH 60‐24 hr	RH 60‐48 hr	RH 60‐72 hr	RH 70‐12 hr	RH 70‐24 hr	RH 70‐48 hr	RH 70‐72 hr	RH 80‐12 hr	RH 80‐24 hr	RH 80‐48 hr	RH 80‐72 hr
Relative crystallinity	2.80 + 0.14d	4.79 + 0.14a	4.93 + 0.24a	3.77 + 0.17b	1.45 + 0.16g	1.94 + 0.09f	2.57 + 0.12de	1.46 + 0.17g	2.01 + 0.04f	2.36 + 0.13e	3.12 + 0.11c	1.49 + 0.07g

Note

Data with different letters in the same column mean significant difference (*p* < .05).

Ambient relative humidity produced a profound effect on the relative crystallinity. At the same storage time, the relative crystallinity first fell and then rose as the RH increased, and it showed the highest value at RH of 60%. Ambient relative humidity enables starch absorbing more moisture and consequently affects the process of starch retrogradation (Johnson & Mauer, 2019). The ample moisture allowed amylose and amylopectin chains moving more flexible, therefore resulting in rearranging of amylose and amylopectin and forming more crystals. However, excess moisture produced a dilute effect, causing the realignment of amylose and amylopectin more difficult, and hence showing a decreased relative crystallinity (Wang, Li, Zhang, Copeland, & Wang, 2016).

### 3.5 Multivariate data analysis

#### 3.5.1 Principal component analysis

All the test methods and their results could determine different influence indicators on sample retrogradation quality, however, these methods did not allow identification of the dominant indicators affecting noodle quality, for example, those contributing most significantly to the retrogradation quality. Therefore, a mathematical method to statistically analyze the data was obtained related to noodle quality (Nwabueze & Anoruoh, 2009). Principal component analysis (PCA) is an effective method to simplify data by dimensionality reduction. According to viewpoint of accumulative variance contribution, principal components with accumulative variance contribution reaching 85% were chosen (Hervé & Williams, 2010).

**Figure 4:**
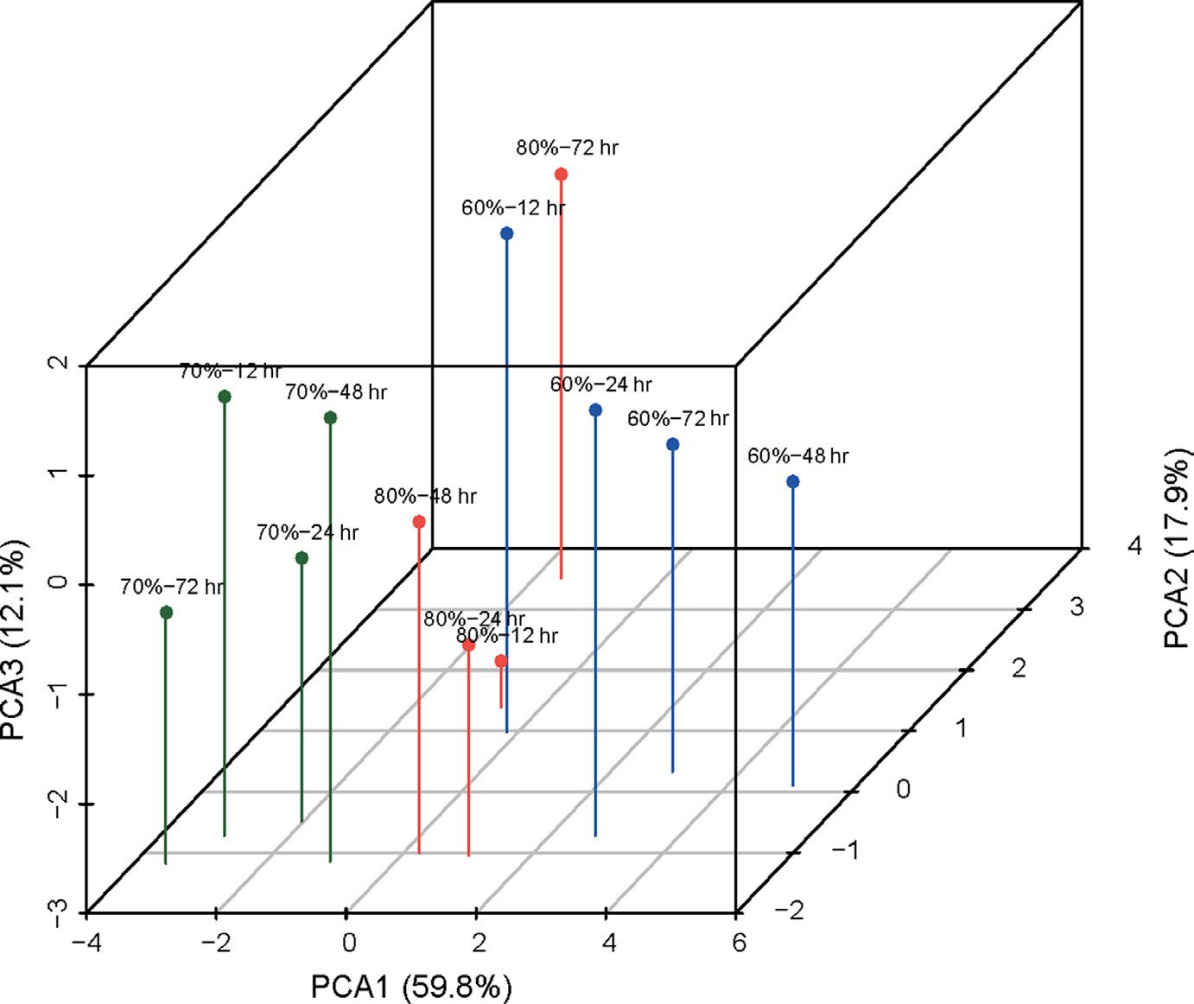
Factor score of different samples based on principal component analysis

Table 4 shows the selected first three principal components whose eigenvalues were greater than 1, and the sum of their information amounted to 89.824% which synthesized most of the information of oat noodles. The contribution ratio of the first principal component (PC1) is 59.819%, and representative indicators are cooking loss, MV, FV, SB, Δ*H*, and relative crystallinity. The cooking loss rate is inversely proportional to PC1, while the remaining indicators are proportional. The contribution ratio of principal component 2 (PC2) is 17.888%, and representative indicators are To and Tp. The contribution ratio of the third principal component (PC3) is 12.117% with the representative indicator Tc. It can be seen from Figure 4 that 12 samples after different retrogradation treatment can be roughly classified into three categories. The first category includes samples of RH60%‐48 hr, RH60%‐72 hr, RH60%‐24 hr, and RH80%‐24 hr covering the positive axis of PC1, suggesting these samples were with less cooking loss, and higher MV, FV, SB, Δ*H*, and relative crystallinity, which contributed better noodle retrogradation quality.

**Table 4:** Component matrix of sample cooking quality and aging quality

Indicator	Component		
	1	2	3
Cooking loss	−0.961	0.112	0.148
PV	0.749	0.219	−0.421
TV	0.979	0.061	0.082
FV	0.984	0.043	0.072
SB	0.959	0.017	0.057
To	0.158	0.925	0.07
Tp	−0.136	0.838	0.425
Tc	0.147	−0.366	0.882
Δ*H*	0.853	0.006	−0.005
Relative crystallinity	0.927	−0.172	0.186
Eigenvalue	5.982	1.789	1.212
Percent of variance (%)	59.819	17.888	12.117

Note

Data with different letters in the same column mean significant difference (*p* < .05).

Samples of RH80%‐48 hr, RH60%‐12 hr, and RH80%‐12 hr could be classified as the second category, which located near the zero scale of PC1, indicating that the cooking loss rate, retrogradation value, and Δ*H* are moderately ranked among the 12 samples. Samples of RH70%‐48 hr, RH80%‐72 hr, RH70%‐24 hr, RH70%‐12 hr, and RH70%‐72 hr were located in the negative direction of PC1, indicating that the five samples have a higher cooking loss, but lower MV, FV, SB, and Δ*H*, therefore, could be classified to the third category. According to the results of the PCA, the test results of To, Tp, and Tc correlated to the extracted principal components were present in relatively low concentrations. They might not be major contributors to the retrogradation qualities of extruded oat noodles.

#### 3.5.2 Cluster analysis

**Figure 5:**
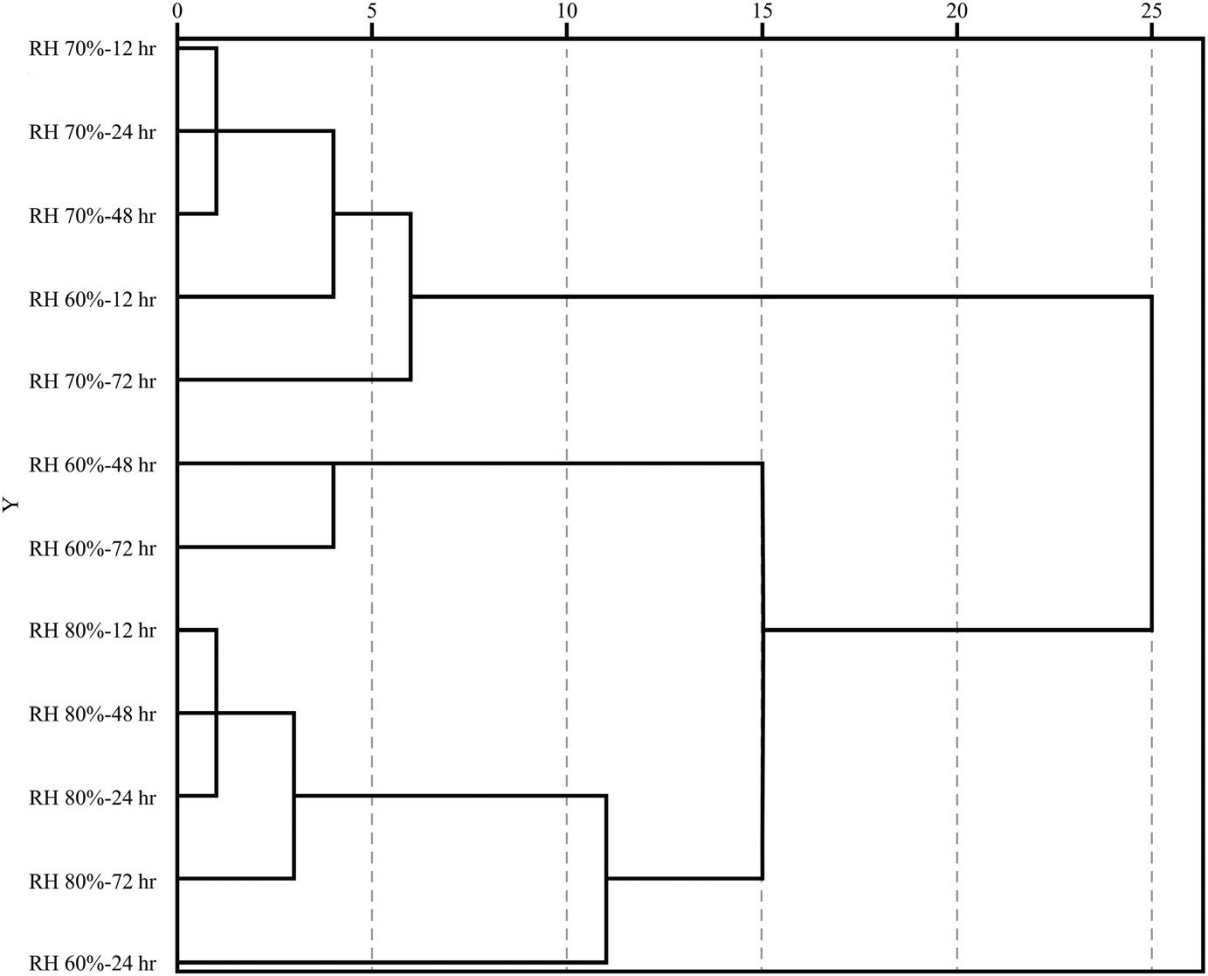
Dendrogram of HCA (hierarchical cluster analysis)

After the ten physical and chemical indicators, data were standardized and converted, the Chebyshev distance was used, and the system was clustered by the squared deviation method (Milligan & Cooper, 1986). The results shown in Figure 5 indicated that four samples under ambient RH 70% and the sample under RH60%‐12 hr gathered in the first category when clustering step was about 6. Samples of RH 60%‐48 hr and RH 60%‐72 hr gathered in the second category when clustering step was about 4. The rest of samples clustered the third category when clustering step was about 3. According to comprehensive analysis, when the relative humidity was 60%, the retrogradation time had a greater influence on the retrogradation quality and cooking quality of the sample, so the samples with different retrogradation time could not be clustered. The samples under relative humidity of 70% and 80% are polymerized into one cluster after different retrogradation time treatments, indicating that retrogradation time had little effect on sample retrogradation quality and cooking quality under this high ambient relative humidity.

When the retrogradation time was 12 hr, samples under relative humidity of 60% and 70% were grouped together, while samples of RH80%‐12 hr did not. When the retrogradation time was 24 hr, samples with relative humidity of 60% and 80% were grouped together without samples of RH 70%‐24 hr. When the retrogradation time was 48 hr and 72 hr, most samples under different relative humidity did not aggregate into one. It shows that different relative humidity had a great influence on the retrogradation quality and cooking quality.

## 4 CONCLUSIONS

Through different retrogradation treatment carried out to extrude oat noodles, the effect of different retrogradation time on noodles has a significant difference on cooking loss, setback value, relative crystallinity, and enthalpy value. As the retrogradation time increases, the cooking quality such as cooking loss tends to decrease first and then increase beyond a certain time. The retrogradation quality such as setback value, relative crystallinity, and enthalpy value first increases and then decreases following similar time period. The higher the degree of retrogradation, the smaller the cooking loss of those noodles. When ambient relative humidity is 60% and the retrogradation time is 48 hr, the cooking loss of extruded oat noodles is the smallest, while the setback value, relative crystallinity, and enthalpy value are the largest. It is indicated that the different retrogradation treatment has significant effects on the cooking quality and retrogradation quality of extruded oat noodles and the optimum retrogradation treatment should be around the ambient relative humidity of 60% and last about 48 hr. The results will provide theoretical guidance for the controlling condition of extruded oat noodle production.

## CONFLICT OF INTEREST

The authors declared that they have no conflicts of interest to this work.
